# Adult Attachment Styles Associated with Brain Activity in Response to Infant Faces in Nulliparous Women: An Event-Related Potentials Study

**DOI:** 10.3389/fpsyg.2017.00627

**Published:** 2017-04-21

**Authors:** Yuanxiao Ma, Guangming Ran, Xu Chen, Haijing Ma, Na Hu

**Affiliations:** ^1^Faculty of Psychology, Southwest UniversityChongqing, China; ^2^Research Center of Mental Health Education, Southwest UniversityChongqing, China; ^3^Institute of Education, China West Normal UniversityNanchong, China; ^4^School of Foreign Languages for Business, Southwestern University of Finance and EconomicsChengdu, China

**Keywords:** attachment style, sensitivity, affective perception, infant facial expression, event-related potentials

## Abstract

Adult attachment style is a key for understanding emotion regulation and feelings of security in human interactions as well as for the construction of the caregiving system. The caregiving system is a group of representations about affiliative behaviors, which is guided by the caregiver’s sensitivity and empathy, and is mature in young adulthood. Appropriate perception and interpretation of infant emotions is a crucial component of the formation of a secure attachment relationship between infant and caregiver. As attachment styles influence the ways in which people perceive emotional information, we examined how different attachment styles associated with brain response to the perception of infant facial expressions in nulliparous females with secure, anxious, and avoidant attachment styles. The event-related potentials of 65 nulliparous females were assessed during a facial recognition task with joy, neutral, and crying infant faces. The results showed that anxiously attached females exhibited larger N170 amplitudes than those with avoidant attachment in response to all infant faces. Regarding the P300 component, securely attached females showed larger amplitudes to all infant faces in comparison with avoidantly attached females. Moreover, anxiously attached females exhibited greater amplitudes than avoidantly attached females to only crying infant faces. In conclusion, the current results provide evidence that attachment style differences are associated with brain responses to the perception of infant faces. Furthermore, these findings further separate the psychological mechanisms underlying the caregiving behavior of those with anxious and avoidant attachment from secure attachment.

## Introduction

Just like the basic needs for food and water in our evolutionary history, the need to be loved and cared for is deeply rooted in individual psychology, especially in infancy. The parent–infant bond, which is central to the human condition, provides the infant with his/her primary social experience (Swain et al., 2014). The quality of interaction between the infant and caregiver is critical for the infant’s survival and development across mammalian species (Swain et al., 2011). Indeed, the caregiving system is a subset of maternal behaviors intending to improve closeness and comfortableness when the caregiver detects internal or external cues associated with situations that she perceives as stressful for the child (Lenzi et al., 2013, 2015). This suggests that the caregiving system is important for organizing and regulating the infant’s emotional, social, and cognitive systems, which is consistent with the accumulating evidence that the quality of caregiving behavior predicts a range of social-emotional child outcomes, including the quality of attachment relationships (Bakermans-Kranenburg et al., 2003; Swain et al., 2014). Moreover, it should be noted that the caregiving system is immature until late adolescence. During puberty, interactions with environmental stimuli, previous attachment experiences, and hormonal and neurobiological changes create a sensitive period that pushes the caregiving system toward maturity (Ammaniti et al., 2000; Grossmann et al., 2006; Lenzi et al., 2013). Through such transformations, late adolescents and young adults, particularly females, show thoughtfulness concerning mothering and they start to present themselves as future parents (George and Solomon, 1999; Lenzi et al., 2013).

The attachment system and caregiving system are complementary systems that are active simultaneously during infant–caregiver interactions (Lenzi et al., 2015). Attachment theory (Bowlby, 1969) postulates that humans are born with a psychobiological system that motivates them to seek proximity to significant others (the mother in particular) in times of need, with the aim of acquiring a feeling of security. Although all infants attach to their caregiver, they manifest different attachment styles depending on the quality of the care they receive. According to Bowlby (1969), the internal working model of attachment is gradually internalized during the interactions between infants and their primary caregiver (usually the mother). When the caregiver is available, responsive, and sensitive and infants are experiencing emotional and physical needs, they tend to manifest patterns of secure attachment. Conversely, chaotic, unpredictable, rejecting, or neglectful care in which non-contingent responses to the child occur frequently lead to an insecure attachment style (Vrtička and Vuilleumier, 2012; Donges et al., 2012). The infant’s current and future behavior is significantly shaped during the infant–caregiver interaction. Early childhood appears to be a critical period for the development of the internal working model of attachment (Leyh et al., 2016b). During this developmental period, neural circuits become organized and adjust to the child’s environment, which is why these neuronal changes are greatly affected by experiences with the attachment figure during the early years (Luby et al., 2012; Whittle et al., 2015). The parent–infant interaction provides infants with their first social environment and forms templates for what they can expect from others and how best to interact with them (Swain, 2008). In addition, there is good empirical evidence for the transmission of attachment security or insecurity from the parental generation to the child (van Ijzendoorn, 1995; Obegi et al., 2004).

Differences in attachment style may predict a selective bias in attention toward certain types of emotional information in the external environment (Edelstein and Gillath, 2008). Caregivers who themselves experience a secure attachment are known to have the ability to perceive the infant’s signals, to interpret them correctly, and to respond to them appropriately (Grossmann et al., 1985; Fraedrich et al., 2010; Leyh et al., 2016a,b). Indeed, the parent’s ability to govern their infant’s distress and fear is crucial to the infant’s ultimate feeling of security (Lenzi et al., 2013). Anxiously attached individuals have a strong need for closeness, worry about relationships, and fear being rejected (Mikulincer and Shaver, 2007; Vrtička et al., 2012). Consequently, such individuals are thought to hyperactivate the attachment system, become highly sensitive and vigilant to potential threat information and devote more cognitive resources to attachment-related material, threat stimuli in particular (Gillath et al., 2005). In contrast to attachment anxiety, in the case of attachment avoidance, proximity seeking is perceived as futile or even dangerous because of the distress felt by failing to achieve proximity to an attachment figure (Vrtička and Vuilleumier, 2012). An avoidantly attached person is marked by compulsive self-reliance and a preferential use of deactivating strategies to keep psychological distance from other people (Vrtička and Vuilleumier, 2012; Vrtička et al., 2012). Accordingly, avoidantly attached individuals tend to have a restricted ability in terms of perceiving and communicating the infant’s feelings and integrating emotional information during parent–infant interaction (Fraedrich et al., 2010). Therefore, the differences in how individuals respond to infants’ needs may be based on individual differences in how they maintain social bonds, namely, their attachment styles.

The present study employed a facial recognition task. Infants’ facial expressions serve as a key component in conveying information about their emotional states and the specific features of infants’ faces contribute to motivate maternal care (Glocker et al., 2009; Leyh et al., 2016a). When investigating the neurophysiological processes of emotional face recognition, different event-related components of the EEG come into play. EEG-ER Ps, a technique used to investigate the temporal brain dynamics of attentive processing at high temporal resolution, was the method employed in current study. The present study focused mainly on the N170 and P300 ERP components. The N170 component is associates with sensitivity to faces and reflects the processing of the configural features of human faces (Eimer and Holmes, 2007; Leyh et al., 2016b). A higher amplitude of the N170 component indicates a higher need for face discrimination resources (Fraedrich et al., 2010; Luo et al., 2016). In addition, top-down control mechanisms operate during the later processing stages and reflect in late ERP responses (>300 ms), which are interpreted as correlates of attention allocation, arousal, control, and/or awareness (Polich, 2007). For example, as mentioned above, the deactivating strategy of an avoidantly attached group was found to be a product of an effortful strategy that occurred rather late in the information processing sequence (Sonnby-Borgström and Jönsson, 2004). Therefore, the P300 component may be particularly useful as it is thought to be a measure of motivated attention and indicates the amount of “motivational relevance” perceived in a stimulus. For example, increases in the motivational relevance of stimuli are reflected in larger P300 amplitudes (Schupp et al., 2000).

People’s attachment styles are also assumed to affect the development and maturation of the brain and have long-lasting effects on brain structures and brain function (Schore, 1994). A recent study focused on the ERPs of mothers during the perception of infants’ emotions by presenting positive, negative, and neutral facial expressions as well as non-facial stimuli within an oddball paradigm (Fraedrich et al., 2010). The study found that insecurely attached mothers demonstrated enhanced N170 amplitudes for the target facial stimuli in the conditions that containing frequent non-facial stimuli. In addition, securely attached mothers showed more pronounced positive P300 amplitudes for target infant faces in the conditions with frequent non-facial stimuli than in the conditions with frequent infant facial stimuli. Furthermore, Leyh et al. (2016a) examined the characteristics of perceptual processing in mothers with different attachment styles by using the oddball paradigm with positive, negative, and neutral infant facial expressions. When the target depicted an infant with negative emotions, insecurely attached mothers exhibited more negative N170 amplitudes than securely attached mothers after the presentation of the target faces with negative emotions, whereas securely attached mothers showed larger P300 amplitudes for the target infant faces than insecurely attached mothers, especially in the conditions with frequent negative infant emotions.

While such recent studies have provided evidence of how attachment-related differences affect the neuropsychological processing of infant facial expressions (Fraedrich et al., 2010; Leyh et al., 2016a), all of the above-mentioned studies have, surprisingly, focused on the difference between securely and insecurely attached groups. However, insecure attachment is more appropriately conceptualized in terms of anxiety and avoidance (Brennan et al., 1998). Differences in anxious and avoidant attachment may predict the perception of, and processing biases toward, certain types of emotional information. Indeed, the behavioral patterns displayed by avoidantly and anxiously attached individuals have been shown to be opposite in almost every respect (Dan and Raz, 2012). Therefore, it is necessary to investigate the neurophysiological processes of infant face recognition in different attachment styles, especially in anxiously and avoidantly attached individuals.

In summary, the purpose of the present study was to test whether participants’ brain responses during the processing of infant facial expressions would be affected by their attachment styles. Based on the findings of previous study (Fraedrich et al., 2010; Leyh et al., 2016a), we proposed the following hypotheses. For the N170 component, we expected the N170 amplitudes of anxiously attached individuals to be significantly larger than those of avoidantly attached individuals in response to all infant facial expressions. Since anxiously attached individuals tend to heighten cognitive accessibility to attachment-related material due to their hyperactivity (Mikulincer et al., 2002; Gillath et al., 2005), while avoidantly attached individuals tend to perform avoidance processes and may reflect less and elaborate less on the emotional experiences they have encoded (Vrtička et al., 2008; Lenzi et al., 2013). Indeed, Zhang et al. (2008) found reduced negative ERP components to faces for avoidantly attached subjects compared to anxiously or securely attached subjects. They suggested avoidantly attached individuals are less elaborative in encoding structural information of faces.

As exemplified by larger P300 amplitudes for securely attached individuals in previous studies (Fraedrich et al., 2010; Leyh et al., 2016a), such individuals may associate with more sensitive caregiving behavior, namely the ability to perceive and interpret the infant’s signals correctly and timely. The larger P300 amplitudes presented in securely attached individuals may reflect an ability to allocate more attentional resources to infant facial expressions and occurs as a result of experiencing more sensitive caregiving behavior themselves.

Regarding the P300 component, securely attached individuals are associated with more sensitive caregiving behavior and the ability to perceive and interpret the infant’s signals correctly, which is exemplified by larger P300 amplitudes for secure attachment, so they can concentrate and allocate more attentional resources to infant facial expressions. Therefore, we assumed that the P300 amplitudes of securely attached individuals would be greater than those of avoidantly attached individuals in response to all infant facial expressions. Moreover, we expected larger P300 amplitudes in anxiously attached individuals than in avoidantly attached individuals in response to crying infant faces because anxiously attached individuals have a strong demand for intimacy, worry about relationships, and fear being rejected, and the crying infant faces may represent a threat to the above-mentioned attributes. At the behavioral level, we assumed that securely attached individuals would have the shortest RTs and anxiously attached individuals would have the longest RTs, this is because the former are more sensitive to infants’ emotional expressions and needs, while the latter may be immersed in the configural processing of the infant faces.

## Materials and Methods

### Participants

We recruited 304 nulliparous female college students to complete the ECR (Brennan et al., 1998) inventory prior to the experiment, which was used to assess the participants’ attachment styles. According to screening criteria (Zilber et al., 2007; Chavis and Kisley, 2012; Dan and Raz, 2012), 65 females met the criteria for experiment. Participants were assigned to three attachment groups according to their score on the anxiety and avoidance subscales of the ECR questionnaire. Participants were categorized in experiment as follows: 20 avoidant attachment; 25 anxious attachment; and 20 secure attachment. All participants were healthy, right-handed, and reported normal or corrected-to-normal vision, and none of them had a prior history of neurological or psychiatric disorders.

### Ethics Statement

The ethics committee of the Southwest University of China approved this study and the recruitment of participants. Written informed consent was obtained from all participants prior to conducting the formal experiment, and all participants gave written informed consent in accordance with the Declaration of Helsinki. All methods were carried out in accordance with the approved guidelines.

### Attachment Scale

The ECR (Brennan et al., 1998) was designed as a dimensional measure of adult attachment styles, including anxiety and avoidance. According to Zilber et al. (2007), Chavis and Kisley (2012), and Dan and Raz’s (2012) criteria, those scoring higher than 1 *SD* above the mean on the anxiety subscale and lower than 1 *SD* below the mean on the avoidance subscale were assigned to the anxiously attached group; those scoring higher than 1 *SD* above the mean on the avoidance subscale and lower than 1 *SD* below the mean on the anxiety subscale were assigned to the avoidantly attached group; those scoring lower than 1 *SD* below the mean on both the anxiety and avoidance subscales were assigned to the securely attached group. The mean and standard deviations of anxiety and avoidant subscale for each attachment group were presented in **Table 1**. The avoidance subscale includes an 18-item scale (α = 0.94) that reflects avoidance of intimacy and interdependence. The anxiety subscale includes an 18-item scale (α = 0.90) that reflects an individual’s concern about rejection and abandonment. Each item is rated on a 7-point Likert scale ranging from 1 (strongly disagree) to 7 (strongly agree). The ECR questionnaire is considered a stable and test–retest reliable (Lopez and Gormley, 2002) measure of an individual’s attachment style.

**Table 1:** ECR scores, and others descriptive statistics of the three attachment groups.

	Total	Secure group	Anxious group	Avoidant group
N	65	20 female	25 female	20 female
Age	21.56 ± 1.64	21.32 ± 0.72	21.71 ± 0.56	21.53 ± 0.83
Anxious score	4.11 ± 0.53	3.01 ± 0.13	5.83 ± 0.18	3.18 ± 0.13
Avoidant score	3.75 ± 0.69	2.88 ± 0.11	3.04 ± 0.14	5.28 ± 0.16


### Stimuli and Procedure

The experimental stimuli consisted of 120 black-and-white photographs of infants aged from 3 to 6 months that were selected from the Chinese Infant Affective Face Picture System (Cheng et al., 2015). The infant facial expressions were either joy, neutral, or crying and 40 pictures were used for each facial expression (50% female). All infant face pictures were identical in size, brightness, contrast, and spatial frequency. Each infant face was presented twice, generating a total of 240 stimuli that were divided into four blocks of 60 trials each. In the experimental session, infant emotions were displayed with equal probability and their presentation was randomized between trials.

**FIGURE 1:**
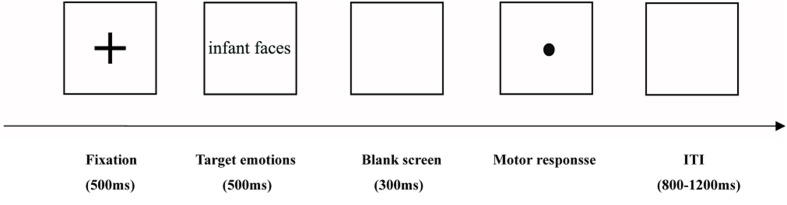
**Schematic illustration of the experimental procedure**.

Participants were seated in a quiet room approximately 80 cm from a computer screen and were required to try their best to avoid eye blinks and head movements. Each trial started with a 500 ms presentation of a black fixation point followed by a 500 ms appearance of the display of the infant face (joy, neutral, or crying). The infant face was then replaced by a 300 ms white blank screen. Following the 300 ms white blank screen display, a black dot was presented on the computer screen. Participants were required to press “1” on the response box with their right hand if the target was a joy infant face, “2” if it was a neutral infant face, and “3” if it was a crying infant face. The dot presentation on the screen was terminated once the participant responded. Following the participant’s response, the screen went blank for an inter-trial interval of 1000 ms with a random jitter of ±200 ms. A schematic illustration of the procedure is shown in **Figure 1**. Prior to the experiment, a short practice block was available to familiarize the participants with the experimental procedure.

### ERP Recording

The EEG was recorded, using the BrainAmp system (Brain Products, Munich, Germany), from 64 scalp sites according to the 10–20 system positions with a reference at FCz (Herrmann et al., 2007). A common average reference was recalculated. Vertical electrooculograms were recorded using electrodes placed below the right eye. Horizontal electrooculograms were recorded from the left and right orbital rim. Electrode impedance was maintained at below 5 kΩ. Signals were amplified using a 0.01–100 Hz band pass filter and continuously sampled at 500 Hz/channel for off-line analysis.

### ERP Analysis

The ERP data were analyzed off-line using the BrainVision Analyzer (Brain Products, Munich, Germany). The EEG activity was separately averaged for correct responses in each condition. All EEG signals were re-referenced off-line on the left and right mastoids (TP9 and TP10). The EEG data were digitally filtered with a 30 Hz low-pass filter and were epoched into a period of 1200 ms (200 ms baseline and 1000 ms post stimulus onset). The analysis of the electrophysiological data focused on selected electrode sites based on previous findings. The N170 component (140–180 ms) was determined over the P5/6, P7/8, and PO7/8 electrodes (Righart and De Gelder, 2008; Ran et al., 2014). Peak amplitudes and latencies of the N170 component were subjected to a repeated-measures ANOVA with attachment style (secure vs. anxious vs. avoidant) as the between-participants factor, and infant expression (joy vs. neutral vs. crying) and hemisphere (right vs. left) as the within-participants factors. Given the absence of a sharply defined peak, the P300 component (300–500 ms after the target stimuli onset) was observed and quantified for mean amplitudes at CP1/CP2, P1/P2, PO3/4, CPz, Pz, and POz (Tacikowski and Nowicka, 2010; Ran et al., 2014). The mean amplitudes of the P300 were entered into a 3 × 3 × 3 repeated-measures ANOVA with infant expression and hemisphere (right vs. middle vs. left) as the within-participants factors and attachment style as the between-participants factor.

## Results

### Behavioral Results

An ANOVA of the correct RTs revealed a main effect for infant expression [*F*(2,124) = 3.91, *p* = 0.023, η^2^ = 0.059], indicating that participants responded faster to crying infant faces (293.92 ± 13.16 ms) than neutral infant faces (306.15 ± 13.24 ms, *p* = 0.041). Although the main effect of attachment style was not statistically significant (*p* > 0.05), a trend was found in which securely attached individuals (285.63 ± 15.64 ms) exhibited the shortest RTs and anxiously attached individuals (319.66 ± 16.85 ms) had the longest RTs (avoidant: 293.12 ± 13.34 ms). There was a significant interaction between attachment style and infant facial expression [*F*(4,124) = 2.46, *p* = 0.049, η^2^ = 0.073], but a simple effects analysis indicated that there were no differences among attachment styles for the infant faces. While the analysis of infant facial expressions within each attachment style showed that securely attached participants’ responses to joy (277.97 ± 24.45 ms) and crying (281.05 ± 23.60 ms) infant faces were faster than to neutral ones (297.87 ± 23.74 ms) (*p* = 0.006 and 0.056, respectively), anxiously attached participants’ responses to crying (306.23 ± 23.60 ms) infant faces were faster than to joy (324.03 ± 24.45 ms) and neutral ones (328.72 ± 23.74 ms) (*p* = 0.032 and 0.012, respectively), while there was no significant difference among infant facial expressions in avoidantly attached participants (joy: 292.99 ± 21.87 ms; neutral: 291.86 ± 21.23 ms; crying: 294.47 ± 21.11 ms). The corresponding ANOVA of the accuracy rates did not yield any significant effects.

### ERP Results

#### N170 Effect

**FIGURE 2:**
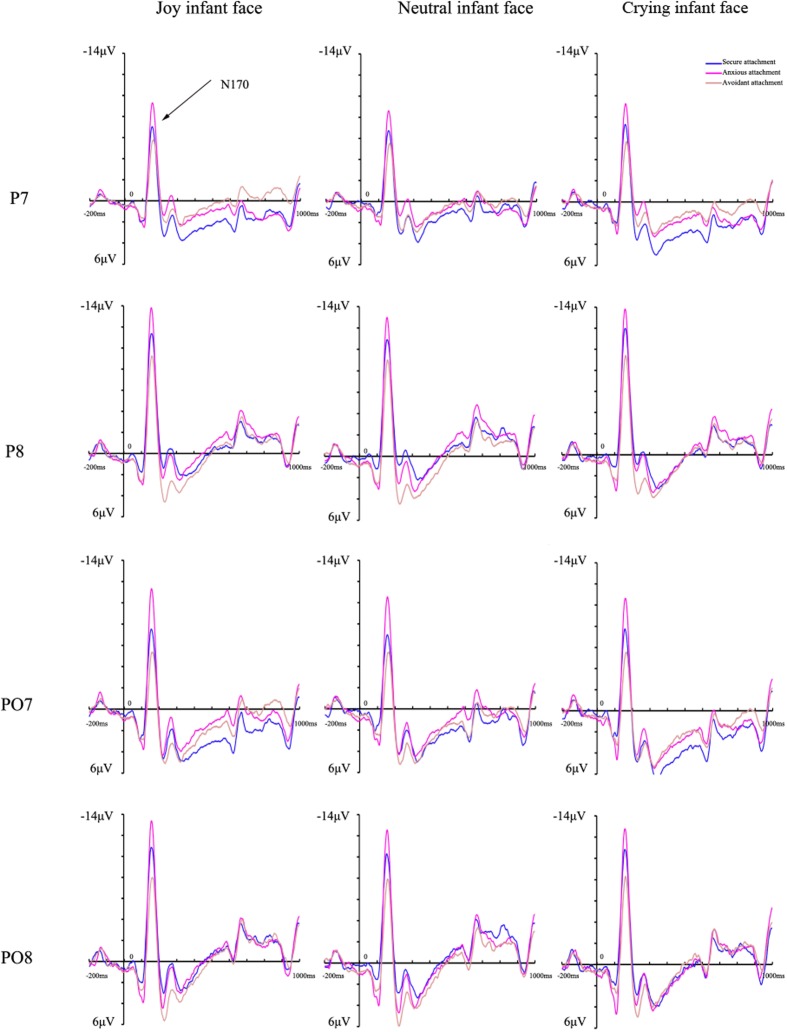
**Grand average event-related potentials of the N170 component recorded at P7/8 and P07/8 in response to joy, neutral, and crying infant faces across attachment styles**.

An ANOVA of the N170 latency revealed a significant main effect of infant facial expression [*F*(2,124) = 17.03, *p* < 0.001, η^2^ = 0.216], reflecting longer latencies for crying infant faces than for joy (155.09 ± 1.20 ms) and neutral (155.04 ± 1.18 ms) ones (both *p* < 0.001). Similarly, the corresponding ANOVA of the N170 peak amplitude revealed a significant main effect of infant facial expression [*F*(2,124) = 5.55, *p* = 0.008, η^2^ = 0.082], with statistically significant larger amplitudes for joy infant faces (-8.23 ± 0.88 μV) than for crying ones (-7.79 ± 0.85 μV) (*p* < 0.001) and marginally significant larger amplitudes than for neutral ones (-7.90 ± 0.86 μV) (*p* = 0.073). More importantly, we found a significant interaction between attachment style and infant facial expression [*F*(4,124) = 2.83, *p* = 0.036, η^2^ = 0.084]. Simple effects analyses indicated that the amplitudes evoked in the anxiously attached group (joy: -10.81 ± 1.41 μV; neutral: -10.18 ± 1.37 μV; crying: -9.80 ± 1.37 μV) were significantly larger than those of the avoidantly attached group that were evoked by all infant faces (joy: -5.72 ± 1.57 μV; neutral: -5.80 ± 1.54 μV; crying: -5.67 ± 1.53 μV) (*p* = 0.019, 0.038, and 0.048, respectively). Additionally, although the amplitudes of the securely attached group for all infant faces (joy: -8.17 ± 1.57 μV; neutral: -7.72 ± 1.54 μV; crying: -7.89 ± 1.53 μV) were at the mid-level between the anxiously and securely attached group, no differences were found in the N170 amplitudes in response to infants’ faces between the securely and avoidantly or anxiously attached group (**Figure 2**). Significant effects related to attachment style were not found for the hemisphere and they are therefore not reported here. No other main effects or interaction effects were observed for latency or amplitude differences for this component.

#### P300 Effect

**FIGURE 3:**
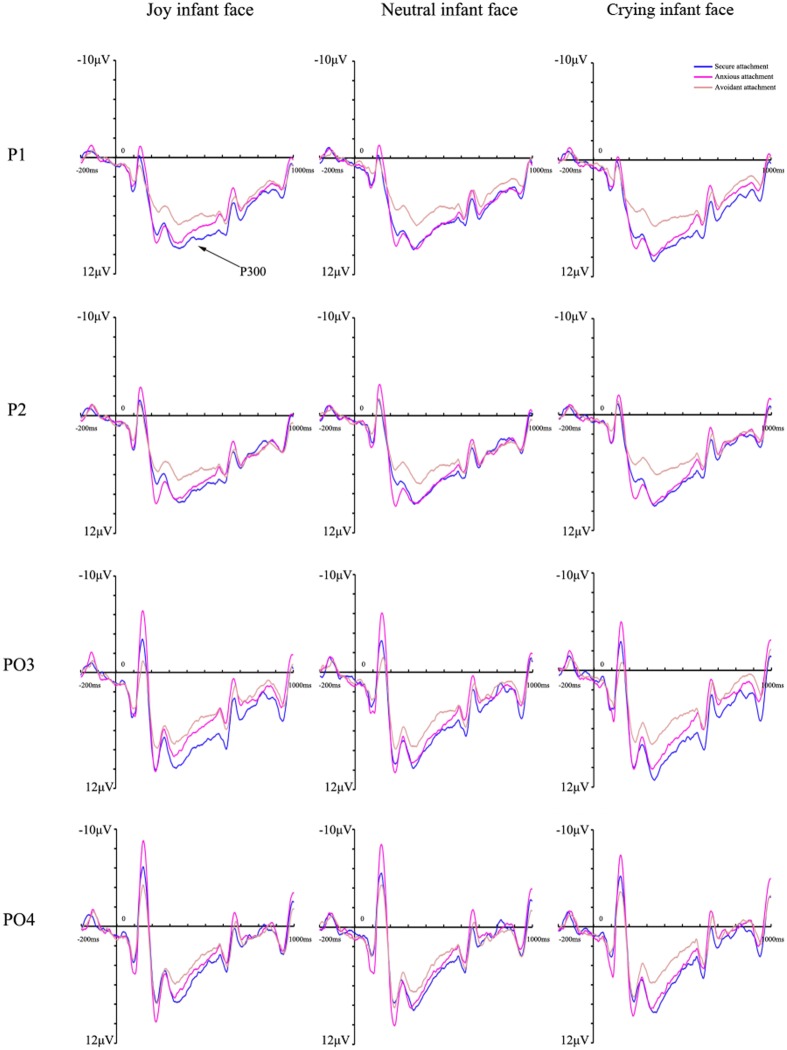
**Grand average event-related potentials of the P300 component recorded at P1/2 and PO3/4 in response to joy, neutral, and crying infant faces across attachment styles**.

**FIGURE 4:**
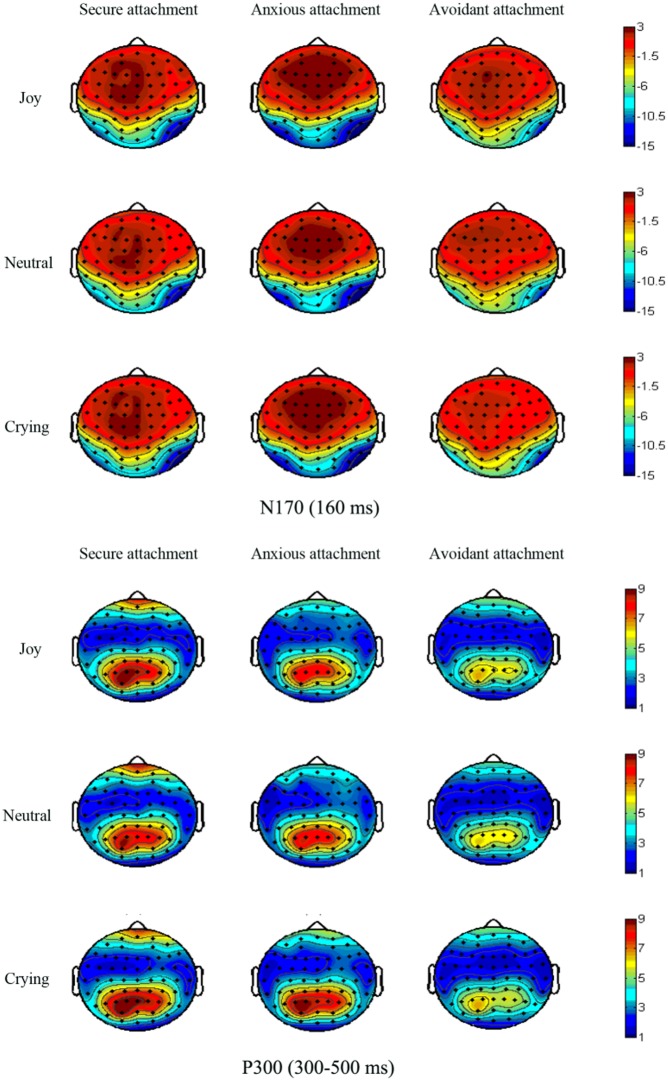
**Topographies of the grand-averaged event-related potentials of the N170 and P300 components in each condition**.

Analysis of the P300 mean amplitudes indicated a significant main effect of infant facial expression [*F*(2,124) = 4.06, *p* = 0.021, η^2^ = 0.062], indicating that the amplitudes for crying infant faces (6.89 ± 0.37 μV) were larger than those for joy (6.64 ± 0.35 μV) and neutral ones (6.58 ± 0.36 μV) (*p* = 0.050 and 0.047, respectively). There was also a main effect of attachment style [*F*(2,62) = 3.66, *p* = 0.031, η^2^ = 0.106], with larger amplitudes being elicited in the securely attached group (7.61 ± 0.63 μV) than the avoidantly attached group (5.34 ± 0.63 μV) (*p* = 0.041). There were no significant differences between the anxiously (7.15 ± 0.57 μV) and securely or avoidantly attached group (*p* > 0.05 for both). Furthermore, the interaction between attachment style and infant facial expression was also significant [*F*(4,124) = 3.14, *p* = 0.019, η^2^ = 0.092], with amplitudes evoked in the securely attached group (joy: 7.59 ± 0.63 μV; neutral: 7.30 ± 0.65 μV; crying: 7.95 ± 0.66 μV) being significantly larger than those of the avoidantly attached group (joy: 5.44 ± 0.63 μV; neutral: 5.36 ± 0.65 μV; crying: 5.23 ± 0.66 μV) for all infant faces (*p* = 0.018, 0.038, and 0.005, respectively). The amplitudes of the anxiously attached group (7.49 ± 0.59 μV) were larger than those of the avoidantly attached group for crying faces (*p* = 0.013). Moreover, we found that the amplitudes evoked by a crying infant face were greater than those evoked by neutral ones in the securely attached group (*p* = 0.006), and the anxiously attached group also exhibited higher amplitudes for crying than for neutral and joy infant faces (*p* < 0.05). No differences were found for the avoidantly attached group (**Figure 3**). Significant effects related to attachment style were not found for the hemisphere and they are therefore not reported here. No other main effects or interaction effects were observed for amplitude differences for this component. The grand average ERP topographies of the N170 and P300 components in each condition are shown in **Figure 4**.

## Discussion

The current study aimed to examine whether the neurophysiological processing of different infant facial expressions by nulliparous females was affected by their attachment styles. On the basis of previous studies, we hypothesized N170 amplitudes of anxiously attached individuals were larger than those of avoidantly attached individuals for all infant emotions; for P300 component, we assumed securely attached individuals would exhibit larger P300 amplitudes than avoidantly attached individuals in all infant emotions, while we expected larger P300 amplitudes in anxiously attached individuals than in avoidantly attached individuals for crying infant faces.

In line with our hypothesis, the ERP results indicated interesting differences between attachment groups. Therefore, specialized neural and cognitive mechanisms that may be involved in the processing of infant faces seem to differ for each attachment style. Regarding anxious attachment, a previous study showed that insecurely attached mothers displayed enhanced N170 amplitudes only if the target facial stimuli were imbedded in frequent non-facial stimuli (Fraedrich et al., 2010). In order to exclude the effect of non-facial stimuli, Leyh et al. (2016a) adopted an oddball paradigm with positive, neutral, and negative infant faces, and found that insecurely attached mothers produced larger N170 amplitudes than securely attached mothers during conditions with a negative target stimulus. However, neither study differentiated anxious attachment from avoidant attachment, both of which were conceived as one central dimension of attachment insecurity. The present research examined further this phenomenon and found that the anxiously attached group exhibited more negative N170 amplitudes than the avoidantly attached group in response to all infant faces. The findings of the N170 amplitudes were consistent with the behavioral results, as the RTs to infant faces of the anxiously attached group were the longest among the three attachment styles. Enhanced N170 amplitudes exhibited by the anxiously attached group might represent a greater need for face discrimination resources (Eimer, 2000) since N170 amplitudes increase when faces are attended to Fraedrich et al. (2010).

Additionally, the anxiously attached group exhibited more positive P300 amplitudes than the avoidantly attached group for crying infant faces, which indicates that a processing bias in the anxiously attached group was only found for crying infant faces. However, what puzzled us is that why a processing bias in the anxiously attached group was not found for joy and neutral faces. As mentioned above, anxiously attached individuals preferentially use hyperactive strategies, so they tend to heighten the effect of attachment-related stimuli due to their hypersensitivity (Mikulincer and Shaver, 2007). Consequently, anxiously attached females devote more cognitive resources to the configural processing of infant faces. For example, several studies have indicated that, at an automatic processing level, anxiously attached individuals are more responsive to emotional facial signals than securely attached individuals (Vrtička et al., 2008; Donges et al., 2012). Therefore, we suspect that this great need for face discrimination resources may lead to less motivational engagement and mental resource allocation to joy and neutral infant faces.

Anxiously attached group has greater P300 amplitudes than avoidantly attached group in crying infant faces. This result is easily understood because anxiously attached individuals are characterized by a strong need for closeness, worrying about relationships, and fearing of being rejected (Mikulincer and Shaver, 2007). It is crucial to focus on threat information, such as a crying infant face, in order to maintain relationships. Previous studies have revealed that anxiously attached individuals exhibit larger P300 amplitudes when the oddballs are angry faces in a background of frequently presented neutral stimuli (Mark et al., 2012) and individuals scoring high on attachment anxiety elicit greater Late Positive Potential amplitudes to negative pictures than those scoring low on attachment anxiety (Zilber et al., 2007). Brain imaging research has also revealed that anxiously attached participants exhibit increased activity in the anterior temporal pole, hippocampus, and dorsal anterior cingulate cortex when thinking about negative emotions, while less activity in the orbitofrontal cortex (OFC) when suppressing such thoughts (Gillath et al., 2005). Similarly, anxiously attached participants show increased activation in the anterior insula and dorsal anterior cingulate cortex during social rejection (DeWall et al., 2012) and a left amygdala response is evoked by angry faces when associated with negative feedback (Vrtička et al., 2008). This evidence also converges with the N170 findings which mirrors the correlation between anxious attachment and higher arousal to negative social clues. However, it should be noted that the P300 result for anxious attachment does not mean that anxiously attached females are sensitive to infants’ signals. As mentioned above, increased attentional resources were found only for crying infant faces because they may represent a threat to the relationship, which is different to secure attachment and the meeting of infants’ needs. Therefore, the processing of crying infant faces in the anxiously attached group may be passive.

The hypothesis that secure attachment would be associated with higher sensitivity to infant faces was supported by our findings, as the securely attached group showed larger P300 amplitudes than the avoidantly attached group for all infant faces. The P300 amplitudes seem to be closely linked to motivational engagement and psychological resource allocation (Duncan-Johnson and Donchin, 1977; Polich and Kok, 1995). Accordingly, heightened P300 amplitudes in securely attached females appear to reflect the allocation of considerably more motivated attention to infant faces. Therefore, securely attached females seem to be better able to perceive infant faces which is a crucial component of sensitivity (Fraedrich et al., 2010). Indeed, previous studies have also suggested that more pronounced P300 amplitudes are evoked by infant faces in securely attached mothers in comparison with insecurely attached mothers (Fraedrich et al., 2010; Leyh et al., 2016a). Moreover, neuroimaging evidence has demonstrated that secure attachment is associated with stronger activation, or increased gray matter volume, in the reward network as well as other interconnected regions, such as the hypothalamus or OFC (Vrtička and Vuilleumier, 2012). For example, securely attached mothers exhibited higher ventral striatum and medial OFC activation than avoidantly attached mothers when seeing images of their own babies (Strathearn et al., 2009). Likewise, as for mothers who score higher on the mother’s positive perception subscale of the Yale Inventory of Parental Thoughts and Actions, there is not only increased gray matter volume, but also more activity in the OFC in response to infant cries (Kim et al., 2010). Indeed, appropriate perception and interpretation of emotion is considered as an essential component of secure attachment (Riem et al., 2012). Therefore, securely attached individuals pay more attention to the infant’s emotional expressions and needs, enabling them to be better at handling their infant’s emotions, which at the same time predicts a high level of sensitivity to the infant’s signals (Spangler et al., 2010).

Avoidantly attached females were associated with a restricted ability in terms of perceiving infants’ faces, as they displayed lower N170 and P300 amplitudes for all infant faces. Indeed, avoidant attachment is linked to a preferential use of deactivating strategies to regulate emotions (Mikulincer and Shaver, 2007), allowing the individual to keep the attachment system in a low activation state and prevent others from perceiving their internal emotional states (Vrtička et al., 2012). For instance, Dan and Raz (2012) found significant differences in the C1 and P1 amplitudes in response to angry compared with neutral faces within only avoidantly attached group. The C1 component (50–100 ms post-stimulus) is the first ERP component triggered by the appearance of a stimulus in the visual field and is thought to be pre-attentive and independent of spatial attention (Stolarova et al., 2006). And the P1 component, an early sensory component peaking around 100 ms post-stimulus, may constitute an index of mobilization of automatic attentional resources (Hopfinger and Mangun, 2001). Therefore, this study indicated that at early perceptual stage, vigilant rather than defensive style is characteristic of avoidant attachment. Moreover, insecurely attached individuals (including three quarters of avoidantly attached individuals) show a larger P1 and attenuated N170 component over the right hemisphere in response to faces (Escobar et al., 2013). Likewise, the neural findings dovetail nicely with the ERP evidence that avoidantly attached individuals activate motor, mirror, and limbic brain areas to a significantly greater extent, but deactivate the medial OFC and the perigenual anterior cingulate cortex (Lenzi et al., 2013). Cecchini et al. (2015) also reported that avoidantly attached individuals showed a correlation with lower activity of the right temporal and limbic areas. Combining these findings mentioned above, we can infer that avoidantly attached individuals might be characterized by a vigilance-avoidance model. Accordingly, avoidantly attached individuals may adopt an avoiding bias in the processing of infant faces (Vrtička et al., 2008; Lenzi et al., 2013), which fits well with the assumptions and empirical findings regarding avoidant attachment.

To date, relatively rare research has examined how adult attachment styles impact the neurophysiological processing of infant faces by nulliparous females. Given the important individual differences in the functioning of emotional processing among attachment styles, and the fact that existing studies did not differentiate anxious and avoidant attachment from insecure attachment, it is worthy of investigating whether the processing of infant face stimuli is affected by individuals’ attachment style, especially in terms of anxious and avoidant attachment. Understanding what role the attachment style plays in the dynamic processing of infant faces can help us to better understand the sensitivity of females with different attachment styles. The current results showed that securely attached females are associated with a high sensitivity to infants’ emotions, whereas anxiously and avoidantly attached females are associated with a low sensitivity to infants’ signals. As infants rely on the quality of the caregiver’s responses to their emotional needs, they may begin to evaluate the emotional expressions of their interaction partners in a certain way and learn to express emotions and behavior in the same way (Fraedrich et al., 2010). Therefore, nulliparous females’ sensitivity during their interactions with infants is crucial as they will play the role of mother in the future. Moreover, it will be interesting to extend our conclusion to non-nulliparous female, as females recruited in present study are college students whose caregiving system is already mature (Ammaniti et al., 2000; Grossmann et al., 2006; Lenzi et al., 2013), but this extension needs further validation. A limitation of the present study is that the ECR questionnaire does not allow the identification of a specific attachment style because it is a dimensional quantitative scale. In future studies, the researchers need to conduct a clinical assessment using the Adult Attachment Interview to identify a specific attachment style. Furthermore, a clinical assessment of the participants should be conducted because the traits of anxiety and depression may also contribute to the perception of infant facial expressions.

Our findings offer a glimpse into the neurophysiological mechanisms involved in the processing of infant faces according to different attachment styles, and especially further uncover the neurophysiological processing characteristics of anxious and avoidant attachment styles for infant faces. As appropriate perception and interpretation of infants’ emotions are considered to be a crucial component of the caregiving system, therefore, different neurophysiological processing of infant faces within the attachment styles may predict females’ varying sensitivity to infants’ signals (Wolff and Ijzendoorn, 1997).

## Author Contributions

YM, XC, GR, and HM performed the data analysis and wrote, reviewed, and approved the manuscript. YM and XC designed the research. NH performed the data collection.

## Conflict of Interest Statement

The authors declare that the research was conducted in the absence of any commercial or financial relationships that could be construed as a potential conflict of interest.

## Abbreviations

ANOVA: analysis of variance
ECR: Experiences in Close Relationships inventory
EEG: electroencephalography
ERPs: event-related potentials
RTs: reaction times
